# Supplementary material for: Effective Optical Image Assessment of Cellulose Paper Immunostrips for Blood Typing
Source: Int J Mol Sci. 2022 Aug 4;23(15):8694. doi: 10.3390/ijms23158694 (PMC9369064; doi:10.3390/ijms23158694)
Supplement: Supplementary file 1 [file ijms-23-08694-s001.zip › ijms-1846348-supplementary.pdf]

International Journal of *Molecular Sciences*

MDPI

*Supplementary Information*

# Effective Optical Image Assessment of Cellulose Paper Immunostrips for Blood Typing

**Katarzyna Ratajczak [1], Karolina Sklodowska-Jaros [1], Ewelina Kalwarczyk [1], Jacek A. Michalski [2,*], Slawomir Jakiela [1,*] and Magdalena Stobiecka [1,*]**

[1] Department of Physics and Biophysics, Institute of Biology, Warsaw University of Life Sciences (SGGW), 159 Nowoursynowska Street, 02776 Warsaw, Poland
[2] Faculty of Civil Engineering, Mechanics and Petrochemistry, Institute of Chemistry, Warsaw University of Technology, Ignacego Łukasiewicza 17, 09-400 Płock, Poland
* Correspondence: jacek.michalski@pw.edu.pl (J.A.M.); slawomir_jakiela@sggw.edu.pl (S.J.); magdalena_stobiecka@sggw.edu.pl (M.S.); Tel.: 48-24-367-2193 (J.A.M.); +48-22-593-8626 (S.J.); +48-22-593-8614 (M.S.).

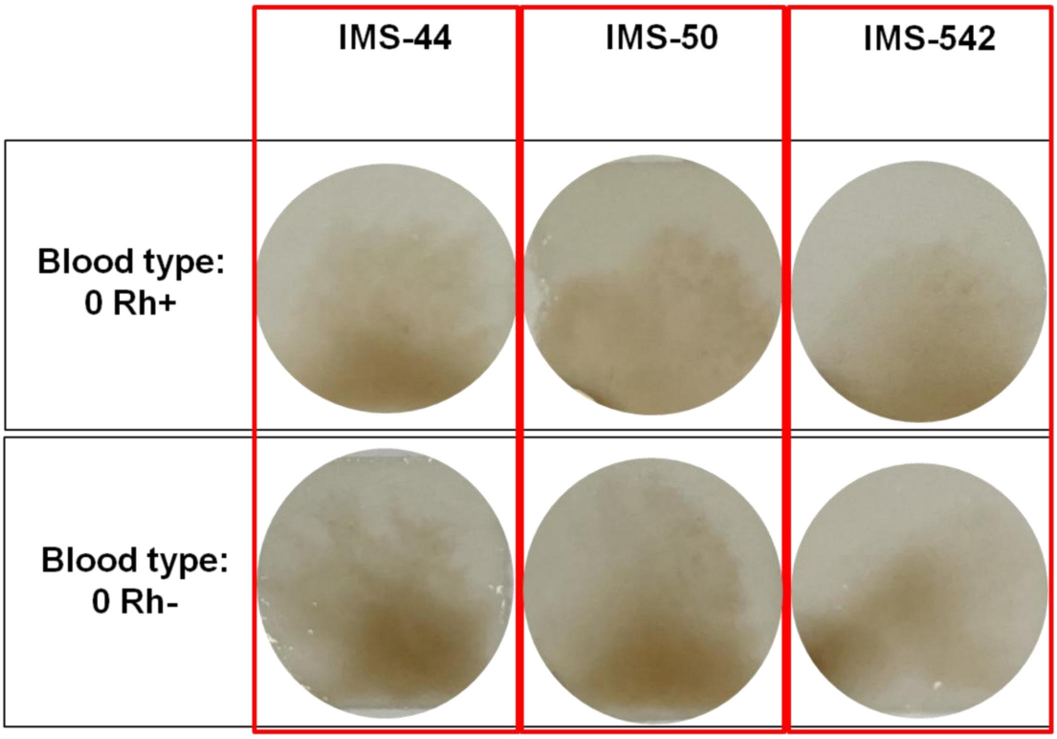


**Figure S1.** Patterns of the negative control performed on uncoated strips on the surface of cellulose membrane IMS-44, IMS-50 and IMS-542 as a result of application of whole blood of different types: 0+ and 0-.

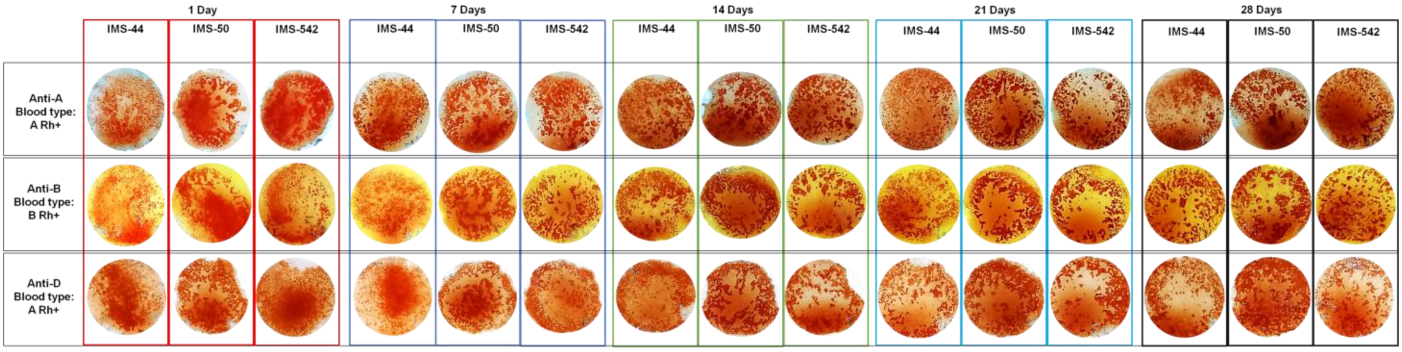


**Figure S2.** Stability of antibodies immobilized on immunostrips IMS-44, IMS-50 and IMS-542 by as a result of interactions of blood of different types: A+, B+ with different kinds of antibodies: anti-A, anti-B, anti-D. All experiments were performed on immunostrips and reagents warmed to room temperature. All immunostrips were stored at room temperature.

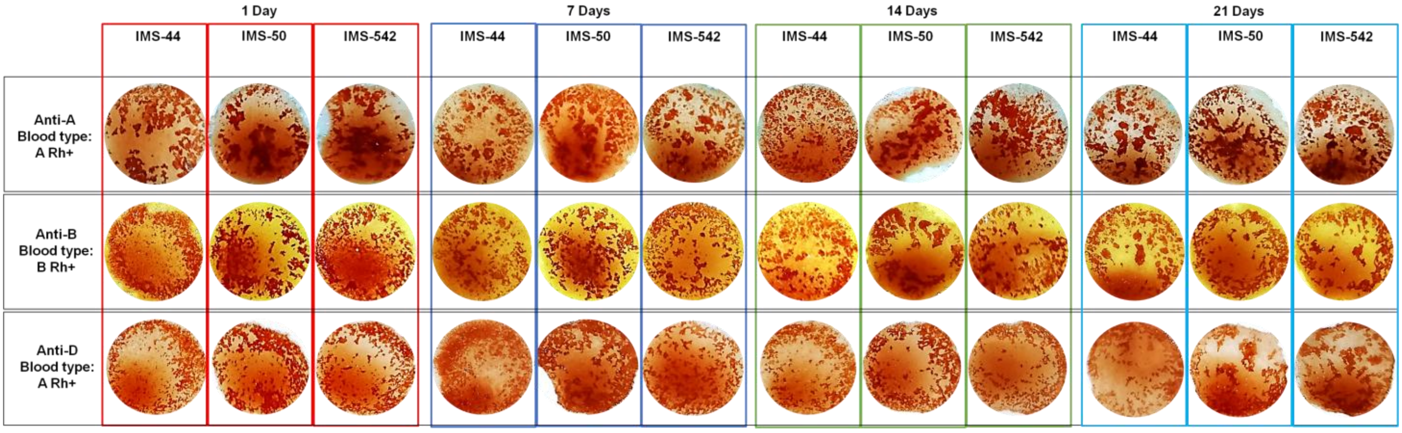


**Figure S3.** Stability of antibodies immobilized on immunostrips IMS-44, IMS-50 and IMS-542 by as a result of interactions of blood of different types: A+, B+ with different kinds of antibodies: anti-A, anti-B, anti-D. All experiments were performed on immunostrips and reagents warmed to room temperature. All immunostrips were stored at 4 °C.
